# Retraction: Suppression of epileptogenesis-associated changes in response to seizures in FGF22-deficient mice

**DOI:** 10.3389/fncel.2023.1279588

**Published:** 2023-08-29

**Authors:** Frontiers Editorial Office

The journal retracts the 18 April 2013 article cited above.

Following publication, concerns were raised regarding the scientific validity of the article due to a duplication in Figure 2A. An investigation was conducted in accordance with Frontiers' policies, and the authors were given the chance to redo the experiments affecting the figure. Editorial assessment of the new data found the SEM values of the new experiments improbable, making the results of the study unreliable. Therefore, this article has been retracted.

This retraction was approved by the Chief Editors of Frontiers in Cellular Neuroscience and the Chief Executive Editor of Frontiers. The authors do not agree to this retraction.

